# Radionuclide Theranostics in Neuroendocrine Neoplasms: An Update

**DOI:** 10.1007/s11912-024-01526-5

**Published:** 2024-04-06

**Authors:** Martina Di Franco, Lucia Zanoni, Emilia Fortunati, Stefano Fanti, Valentina Ambrosini

**Affiliations:** 1 Nuclear Medicine, Alma Mater Studiorum, University of Bologna, Via Massarenti 9, 40138 Bologna, Italy; 2 Nuclear Medicine, IRCCS, Azienda Ospedaliero-Universitaria Di Bologna, Bologna, Italy

**Keywords:** Neuroendocrine tumors, NET, PET, PRRT, Theranostics

## Abstract

**Purpose of Review:**

This paper aims to address the latest findings in neuroendocrine tumor (NET) theranostics, focusing on new evidence and future directions of combined diagnosis with positron emission tomography (PET) and treatment with peptide receptor radionuclide therapy (PRRT).

**Recent Findings:**

Following NETTER-1 trial, PRRT with [177Lu]Lu-DOTATATE was approved by FDA and EMA and is routinely employed in advanced G1 and G2 SST (somatostatin receptor)-expressing NET. Different approaches have been proposed so far to improve the PRRT therapeutic index, encompassing re-treatment protocols, combinations with other therapies and novel indications. Molecular imaging holds a potential added value in characterizing disease biology and heterogeneity using different radiopharmaceuticals (e.g., SST and FDG) and may provide predictive and prognostic parameters. Response assessment criteria are still an unmet need and new theranostic pairs showed preliminary encouraging results.

**Summary:**

PRRT for NET has become a paradigm of modern theranostics. PRRT holds a favorable toxicity profile, and it is associated with a prolonged time to progression, reduction of symptoms, and improved patients’ quality of life. In light of further optimization, different new strategies have been investigated, along with the development of new radiopharmaceuticals.

## Introduction

**Fig. 1:**
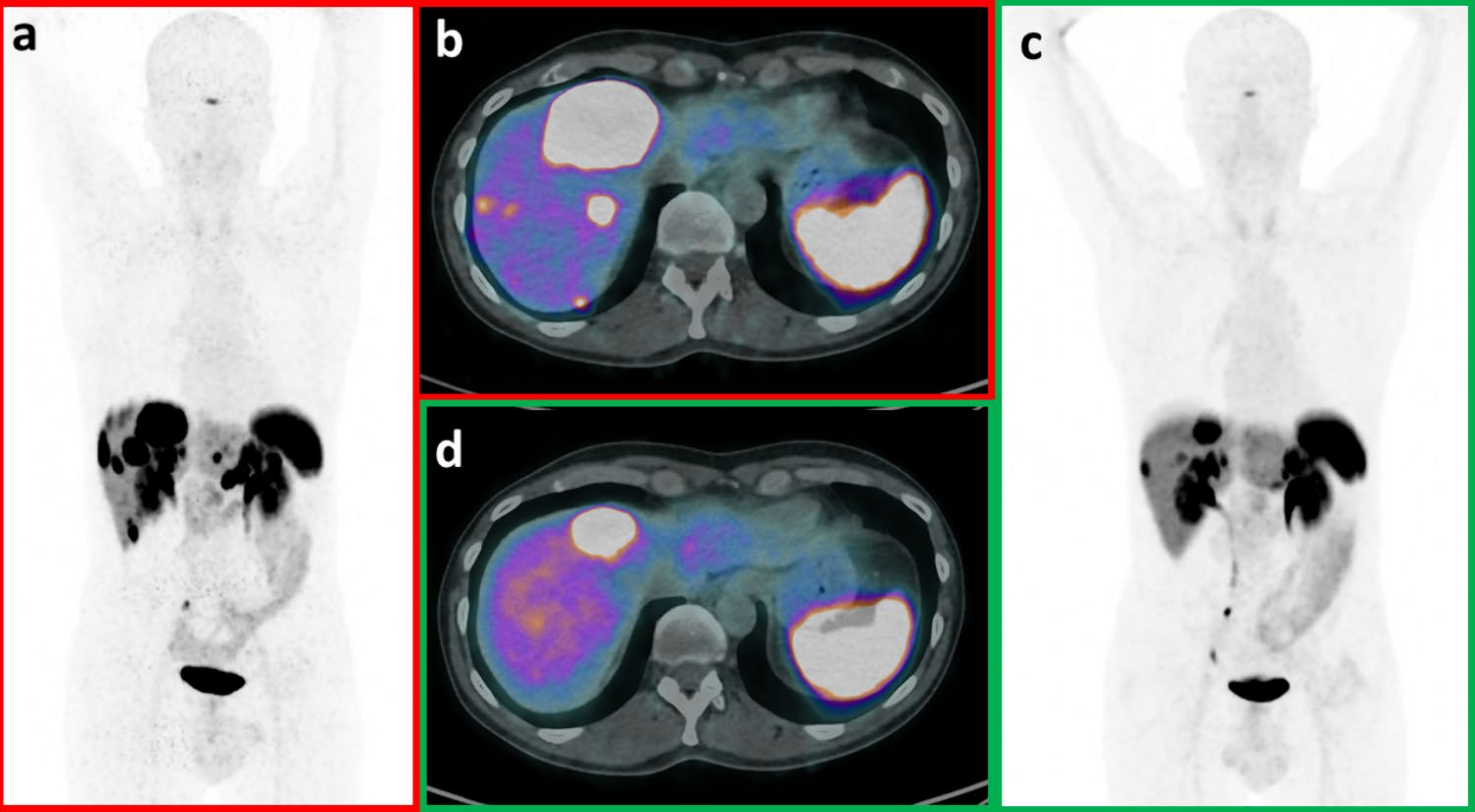
Patient with pancreatic NET G1 (Ki-67, 1.7%) treated with 4 cycles of PRRT with [177Lu]Lu-DOTATATE. Baseline [68 Ga]Ga-DOTANOC PET/CT (**a**, **b**) showed high uptake in multiple liver lesions. [68 Ga]Ga-DOTANOC PET/CT after PRRT (**c**, **d**) documented reduced lesions’ number and reduced intensity of uptake, in keeping with partial response to PRRT

Theranostics is one of the most promising applications of precision medicine and a major emerging branch of nuclear medicine. It relies on the concept of targeting disease-specific features to detect and treat tumor lesions (Fig. 1): at first, a radiopharmaceutical is labeled with a positron-emitting radionuclide and used for positron emission tomography (PET) lesion detection, then the same compound is labeled with a different radionuclide, emitting a cytotoxic radiation (e.g., beta-minus or alpha) for target treatment [1].

The forerunner application of the theranostic approach was the use of radioactive iodine for diagnosis and treating thyroid disease in 1950s [2]. Neuroendocrine neoplasms (NEN) have been diagnosed and treated with radionuclides for over 20 years and are referred today as a paradigm for modern theranostics [3].

NEN are rare malignancies that can arise anywhere in the human body and can be functioning (if the secretory activity is maintained) or, more frequently, non-functioning (detected incidentally or following tumor-mass symptoms). The most recent 2022 WHO Classification of Endocrine Tumors divides NEN according to the cell of origin into neural and epithelial types; the latter are sub-categorized depending on their cells’ differentiation grade in well differentiated, i.e., neuroendocrine tumors (NET) and poorly differentiated, i.e., neuroendocrine carcinomas (NEC) [4]. Ki67 further classifies NET in G1 (< 3%), G2 (3–20%), and G3 (> 20%); NEC are high-grade by definition (ki-67 > 20% but generally > 55%). This grading system provides prognostic information: the higher the grade, the worse the prognosis [4].

NET originate more frequently from the gastro-entero-pancreatic tract (GEP-NET), followed by the lung (typical and atypical forms), with incidence rates respectively of 3.56 and 1.49 per 100,000; other localizations are less frequent [5]. NET incidence, and in particular NET prevalence, are increasing, following earlier diagnosis and new treatment strategies leading to improved survival rates [5].

Although frequently heterogeneous in terms of primary tumor site, clinical presentation, and behavior over time, NET are typically slow-growing and usually express somatostatin receptors (SST), mostly type 2. This feature has been used to detect tumor sites with SST PET with computed tomography (PET/CT) and to selectively treat SST-expressing lesions with peptide receptor radioligand therapy (PRRT) with [6]Y- or [177]Lu-labeled somatostatin analogs [1].

PRRT with [177Lu][Lu-DOTA0-Tyr3]octreotate ([177Lu]Lu-DOTATATE) was approved by the European Medicines Agency (EMA) in 2017 and Food and Drug Administration (FDA) in 2018 [7]. According to EMA, [177Lu]Lu-DOTATATE is approved for unresectable or metastatic, progressive, well-differentiated (G1 and G2), SST-positive GEP NET in adults [7]. Patients’ eligibility for PRRT requires SST PET/CT positivity (uptake considered significant if at least above the liver parenchyma) [8]. PRRT is included in ESMO and ENETS clinical guidelines [9, 10].

The aim of this paper is to critically highlight the latest issues concerning NEN theranostics.

## Updates in PRRT

### New Evidence on Safety and Efficacy

NETTER-1 was a phase III randomized, multicentric, open-label trial which aimed to evaluate the safety and efficacy of PRRT in patients with progressive well-differentiated and SST-positive midgut NET. Progression-free survival (PFS) was longer in the 229 patients of the study arm (who received 4 cycles of 7.4 GBq of [177Lu]Lu-DOTATATE every 8 weeks and 30 mg octreotide LAR every 4 weeks) vs the control arm (FDA-approved off-label use of high-dose octreotide LAR every 4 weeks) [11]. The final results showed a longer median overall survival (OS), for the PRRT arm (48 months vs 36.3 months), although not statistically significant, due to a high rate of cross-over of the patients in the control arm [12••]. In a deeper analysis adjusting survival of those patients who crossed-over to PRRT (36%), the adjusted median OS was 30.9 months in the control group [12••]. The Netter 1 trial showed that PRRT is safe: final results showed that in the whole study, 7/111 (6%) patients had grade > 3 treatment-related toxicity, i.e., nephrotoxicity, grade > 3, 5%; hematological toxicity, 2%, myelodisplastic syndrome (MDS) [12••].

Gordon et al. recently published an analysis of their 5-year experience in administering [177Lu]Lu-DOTATATE after its approval. They treated 143 patients with advanced NET, administering ~ 8 GBq for 4 cycles every 8 weeks, and observed a median PFS of 32.3 months, a median OS of 72.5 months, and no evidence of renal toxicity, while MDS occurred in 5% of the subjects [13]. Favorable results were also reported in Brazilian (*n* = 36; mPFS = 23.0; OS = 30.0 months for G3 patients) [14], Polish (*n* = 167; mPFS = 29.3; OS = 34.0 months) [15], and Korean (*n* = 64; mPFS = 21.7; OS not reached within the 15.7-month follow-up) [16] cohorts.

PRRT was also reported to be safe in the elderly (Theiler et al. described a comparable safety profile in young and elderly patients aged > 79) [17] and in patients with chronic kidney disease [18].

In a large cohort of 1631 patients who received PRRT from 1999 to 2019, Chantadisai et al. reported therapy-related myeloid neoplasms (t-MN) in 30 cases only (1.8%), with a median latency period (from first PRRT to diagnosis of t-MN) of 43 months (range 6–123). The median OS from diagnosis of t-MN was 13 months (all patients), 6 months in the acute myeloid leukemia subgroup (seven patients), and 15 in the MDS subgroup (23 patients) [19].

Although PRRT was reported as a safe therapeutic option in NET patients with up to 50% liver involvement [20–22], a more recent study by Gococo-Benore et al. investigated the risk of radiation-induced hepatotoxicity in patients with more than 75% of liver involved. Of 15 patients included (371 screened), only one experienced hyperbilirubinemia (grade 1 hepatotoxicity) and no one went through grade 3–5 hepatotoxicity, highlighting the potential feasibility of PRRT also in high-burden liver disease [23•].

Overall, the most recent studies confirm the knowledge acquired so far regarding PRRT safety and efficacy and show comparable outcome data, demonstrating that this therapeutic option is founded on solid evidence [9, 10].

### Retreatment

Given the limited therapeutic options for progressive GEP-NEN and the known frequent adverse events caused by sunitinib and everolimus, retreatment with [177Lu]Lu-DOTATATE (R-PRRT) has been considered for NET patients who progressed after receiving first course PRRT (I-PRRT), with either [177Lu]Lu-DOTATATE or 90Y-PRRT. In a meta-analysis by Strosberg et al., including 13 studies investigating R-PRRT (comparable indications), a similar safety profile was observed in comparison with I-PRRT (grade 3–4 toxicity of any type in 5% of patients, hematologic toxicity of grade 3–5 in 9%). The estimated PFS was > 12 months in all the sub-groups, with a DCR (disease control rate) of 70%, but OS was not reported in all included studies [24]. OS data were available in the studies by Severi et al. (*n* = 26; median OS after retreatment = 36 months), Rudisile et al. (*n* = 35; median OS after I-PRRT not reached within a follow-up of 71 months, median OS after retreatment = 51 months), and Van Der Zwan et al. [25–27]. Van Der Zwan et al. investigated the largest sample (*n* = 181) of retreated patients with bronchial or GEP-NET, receiving a single radiopharmaceutical ([177Lu]Lu-DOTATATE) and up to three treatment courses: patients were selected for re-(re)treatment if they had benefited from I-PRRT with a minimal PFS of 18 months. Patients received an additional cumulative dose of 14.8 GBq of [177Lu-DOTA,Tyr3]octreotate over two cycles per retreatment with R-PRRT or re-retreatment with PRRT (RR-PRRT). PFS was 19 months across all primaries. Combined OS after I-PRRT plus R-PRRT and RR-PRRT was 80.8 months (95% CI 66.0–95.6) [27].

Across studies investigating re-treatment, all courses consisted of 1 to 6 cycles and the doses administered were variable, but in 6/7 works where [177Lu]Lu-DOTATATE was employed for both I-PRRT and R-PRRT, they were superimposable, i.e., 7.4 GBq per-cycle [26–29].

More recent studies confirmed the safety profile of R-PRRT and a PFS > 12 months: in a retrospective single-center study, Sitani et al. observed an acceptable safety profile (no grade 3–4 toxicity) and a PFS of 17 months after R-PRRT in a population of 22 re-treated patients, while the median OS was not reached during the follow-up period of 72 months [30•]. Silva et al. reported a median PFS of 17.5 months (IQR 7–39) for PRRT re-treated patients (*n* = 22), longer PFS after 2 cycles vs 1 and a median OS from I-PRRT of 66 months (IQR 65–90), without significant toxicity except for a lowering of platelet count (grade 1 toxicity, 10 patients, *P*-value = 0.055) [31].

Interestingly, Rodrigues et al., in addition to the outcome data of 40 patients treated with a second course of PRRT (PFS = 19.37; median OS = 122), analyzed the predictive value of [18F]FDG PET/CT status before and after the first and the second PRRT course. They found a more favorable outcome after the second PRRT course in the FDG-negative group (*n* = 26; median OS = 145.5 months; 95% CI 83.34–207.67) than the FDG-positive group (*n* = 14; median OS = 95.06 months; 95% CI 48.36–141.77) [32].

Across the different studies, the minimum PFS from the first PRRT, required as re-treatment eligibility criteria, was very heterogeneous, i.e., a PFS after the initial PRRT of > 12 months, ≥ 6 months, and no fixed time, respectively, in the works of Sitani et al., Silva et al., and Rodrigues et al. [30•, 31, 32]. Criteria for choosing a time-interval over another were not proposed in any of the studies.

Other limitations of the available works include the retrospective study designs, different treatment response criteria, and the heterogeneous populations in terms of initial treatment (radiopharmaceutical/doses/number of cycles), variability of time to progression (TTP), and intervening therapies from the first PRRT.

The multicenter randomized controlled trial ReLUTH, still recruiting, is the first prospective trial evaluating PRRT retreatment. It aims to compare two different [177Lu]Lu-DOTATATE schemes of retreatment (2 + 2 cycles vs 2 cycles) in patients with intestinal NET progressing after 12 months from the first PRRT course, expecting that four cycles will be more effective than two, with limited adverse impact on safety [33].

In conclusion, preliminary results are supporting PRRT retreatment as a feasible option in patients progressing after I-PRRT. R-PRRT is expected to be particularly effective in cases presenting a good response to I-PRRT, with a similar limited toxicity profile with respect to the first course. Randomized controlled trials are needed to directly compare the survival benefit of PRRT retreatment to other therapy lines like sunitinib or everolimus.

### The Issue of Response Assessment

Providing adequate evaluation of PRRT response in patients with advanced NET remains an unsolved problem. Criteria used so far for response evaluation of solid tumors, i.e., Response Evaluation Criteria in Solid Tumors 1.1 (RECIST 1.1) [34], mRECIST [35], or WHO [36], are based on dimensional changes and are suboptimal for NET distinctive slow-growing lesions. Moreover, patients with metastatic NET frequently do not display a morphological response to systemic therapies, including PRRT, that frequently results in disease stabilization [37]. It has been demonstrated that preventing disease progression, either through stabilization or obtaining remission (a concept referred to as “disease control rate” (DCR)), produces comparable outcomes in NET patients [38]. Although not optimal, morphology-based response criteria are currently used in clinical trials because they provide a standardized framework for objective assessments. Conventional imaging is routinely used in clinical practice to document progression and subsequently start further-line treatments in advanced NEN. Therefore, contrast enhanced computed tomography or magnetic resonance imaging remains irreplaceable as their spatial resolution allows for the detection of small dimensional changes that may be missed by PET.

On the other hand, PET functional imaging may detect even non-enlarged nodes or bone lesions (either millimetric or without corresponding morphological changes on CT) when presenting high SST expression. In addition, mere changes in tumor dimension may lead to equivocal interpretation. For example, a stable lesion may mask intralesional de-differentiation while a decrease in tumor volume after chemotherapy could subtend a cyto-reductive effect on the most aggressive components without a significant effect on SST-expressing cells. In addition, an initial increase in tumor volume may be related to inflammatory mechanisms (i.e., pseudoprogression), involving up to 10% of patients after PRRT [39].

For this reason, a complementary evaluation by conventional imaging and SST PET/CT, and, when necessary, [18F]-fluorodeoxyglucose ([18F]-FDG), has been suggested to evaluate both dimensional changes and functional phenotypes. Nevertheless, in the EANM Focus 3 meeting, consensus was not reached on the preferential imaging technique to assess PRRT response [40].

In a recent retrospective study, Zwirtz et al. compared Choi, RECIST, and SST-PET/CT criteria in 34 patients who received at least two PRRT cycles. EORTC [41] was the chosen model for somatostatin-based evaluation, named MORE and based on maximum standardized uptake value (SUVmax). The authors conceptualized a new parameter, ZP, defined as the product of SUVmean and Hounsfield Units (HU) of target lesions. PET/CT was performed at baseline and after every PRRT cycle (mean of 3.2 months). Patients progressing after the second PPRT cycle according to PET-based criteria (MORE and ZP) had a shorter survival compared to responders, but not statistically significant, and OS was not reached. The authors observed significant variability between all different criteria, uncovering the weakness of current evaluation systems and suggesting further investigations of PET-based parameters for response assessment [42•].

Molecular imaging allows to obtain functional information on whole tumor burden that is not achievable otherwise, i.e., tumor volume parameters, whose potential value for response assessment is under investigation and will be discussed in the next chapter.

In conclusion, the formulation of NET-specific response assessment criteria constitutes an ambitious challenge and an unmet need.

### PET Parameters for Predictivity and Prognosticity

#### SST-PET/CT

In the last decade, SST-PET/CT has almost completely replaced somatostatin receptor scintigraphy as a tool to select patients for PRRT, due mostly to its higher accuracy and quantification.

Previous analysis focusing on the predictive value of SUV before and/or after PRRT provided different results [43–47] and was carried out in mostly heterogenous cohorts (in terms of primary tumor site), often analyzed using different response criteria and assessing different functional parameters. In a meta-analysis by Lee et al. [48], eight studies showed that a higher baseline SUV was associated with favorable outcomes: it was predictive of responding lesions (using SUVmax [49–51]; SUVmax average in up to five target lesions [51]; tumor-to-spleen ratio, SUVT/S [49]; tumor-to-liver ratio, SUVT/L [49, 50]); of longer OS (using SUVmean [52]); of longer PFS (using SUVmax [50, 51, 53], SUVT/S and SUVT/L [50]); of reduced lesion diameter (using SUVmax [54]).

Overall, a low SUVmax on baseline PET/CT was associated with a worse outcome and a high baseline SUV predicted response [55] due to the high citotoxic effect on the over-expressed SST target. The same rationale can explain why a decrease in SUVmax after PRRT was reported in responders [54, 56–58] and a decreasing ΔSUV from baseline to follow-up was associated with longer PFS [51] and longer time to progression using ΔSUVT/S [43]. However, caution is needed in interpreting SUV decrease as a predictor of low risk of progression, because the de-differentiation phenomena can cause a lowering of SST expression as well.

Tumor-to-blood ratio (TBR), defined as the ratio between the SUVmean of the tumor divided by the SUVmean of the left ventricle or aortic blood pool, was found to correlate to NET cells’ Ki67 and to reflect SST expression better than SUVmax [59]. Following this rationale, Weber et al. recently investigated the predictive value of TBR (SUVmean of the tumor were divided by the SUVmean of the left ventricle) in a retrospective single-center analysis of 139 individuals diagnosed with NET, who received PRRT and underwent baseline and follow-up PET/CT. Patients with baseline TBR in the 1st quartile had a shorter PFS (14.4 months) than those in the 3rd (23.7 months; *P* = 0.03) and 4th (24.1 months; *P* = 0.02) quartile. Also, these patients had significantly shorter OS (32.5 months) than those with baseline TBR in the 2nd (41.8 months; *P* = 0.03), 3rd (69.2 months; *P* < 0.01), and 4th (42.7 months; *P* = 0.03) quartile. Baseline to follow-up increases in TBR were independently associated with shorter PFS and OS while changes in SUVmean were not [44].

Other recently investigated parameters are tumor volumes, mostly total receptor volume (RTV), referring to the summed SST-volumes of the segmented lesions, and total lesion activity (TLA), calculated by the sum of the product of each lesions’ SUVmean and its corresponding SST-expressing volume.

In a prospective study with one of the largest populations, Tirosh et al. enrolled 184 NET patients who underwent SST PET/CT before receiving PRRT. PET parameters including RTV were calculated. The authors found a significant association between RTV and risk for disease progression (for RTV ≥ 7.0 mL) on univariate (*P* = 0.02) and multivariate analyses (HR 3.0, 95% CI 1.1–8.7, *P* = 0.04). Moreover, RTV ≥ 35·8 mL was associated with a higher disease-specific mortality on multivariate analysis (HR 10.6, 95% CI 1.6–68.9, *P* = 0.014) [58]. Further recent studies confirmed the correlation between baseline RTV and worse prognosis in terms of PFS or TTP and OS [60–63].

A meta-analysis conducted by Hou et al. (nine studies and 593 patients) reported an association between high RTV and shorter PFS and OS [64].

Mileva et al. recently reported the results of the prospective phase II LUMEN study, in which they used different imaging modalities (SST-PET/TC, FDG-PET/CT, and MRI) at baseline and after the first PRRT cycle in 37 patients. Early documentation of a RTV decrease of more than 10% in SST-PET/CT after the first cycle was associated with longer PFS (51.3 vs. 22.8 months; hazard ratio, 0.35; 95% CI, 0.16–0.75; *P* 5 0.003) [37].

The limitations of the studies conducted so far mostly regard small cohorts and the inclusion of NET of different primary sites, known to have different clinical behaviors and outcomes after PRRT, as shown in the recently published results of the SEPTRALU trial [65•].

In summary, clinical prediction of PRRT response based on baseline SST-PET parameters is still under investigation.

#### [18]FDG PET/CT

Intralesional heterogeneity may be a marker of poorer prognosis. In a recent study by Graf et al., SST intralesional heterogeneity was assessed as visually interpreted changes in the modified Krenning score across a single target lesion. A change from grade 3–4 to grade 2 or from grade 2 to 1 in 3 dimensional analysis within a lesion (> 5 mm in all axes) was used as the cutoff to define a lesion as heterogeneous. In cases of central necrosis, SST expression was assessed on the periphery. Patients were classified as having heterogeneous SST expression if > 50% of their target lesions were heterogeneous: this group showed a lower median TTP (26 months vs 54 months of cases presenting homogeneous lesions). In this patient cohort, visual assessment of SST-heterogeneity was both predictive and prognostic [66].

FDG is an indirect indicator of NEN lesions’ heterogeneity due to its ability to identify undifferentiated clones with typically low SST that are expected to drive disease progression. However, FDG is generally not considered as a routine component of the pre-PRRT diagnostic flow-chart, although it is widely performed in many centers. It is well known that patients with G3 or high-grade G2 tumors may exhibit some FDG-avid lesions and that even patients originally graded as G1 at first pathological assessment may develop FDG-avid clones at disease progression [67, 68]. Patients presenting spatially concordant FDG/SST avid lesions are expected to show reduced PRRT efficacy. On the contrary, spatially mis-matched FDG/SST lesions represent a contraindication to PRRT alone. In a recent meta-analysis encompassing 12 studies and a cohort of 1492 patients across all tumor grades, researchers found that patients with negative FDG scans prior to PRRT exhibited a superior disease control rate, as well as extended PFS and OS [69].

Binderup et al. prospectively enrolled 166 patients of all grades (median follow-up time of 9.8 years): FDG-positivity was associated with a shorter OS compared with a negative scan (hazard ratio (HR), 3.8; 95% CI, 2.4–5.9; *P* < 0.001). Moreover, in G1/G2 patients (*n* = 140), FDG-positivity was the only identifier of high-risk for death (HR, 3.6; 95% CI, 2.2–5.9; *P* < 0.001), demonstrating its superiority over histologic grading for risk stratification [70].

In a retrospective study by Zhang et al. encompassing a large population of patients treated with PRRT (*n* = 495), FDG PET resulted as an independent prognostic factor. FDG positivity was associated to shorter median PFS (18.5 months vs. 24.1 months) and OS (53.2 months vs. 83.1 months) [71].

Given the high prognostic value of FDG PET, different grading systems have been proposed to standardize the interpretation of the double tracer SST/FDG imaging [67, 72]. Among them, the NETPET score by Chan et al. (P0 = normal scan on both tracers; P1 = presence of SST positive/FDG negative lesions only; P2–4 = positivity on both tracers, with gradual escalation in FDG uptake; P5 = presence of FDG-positive/SST-negative lesions only) [67] was validated by a multicenter study confirming its prognostic role (median OS/TTP of 101.8/25.5 months for P1, 46.5/16.7 months for P2–4, and 11.5/6.6 months for P5) [73].

A recently published multicenter study by Chan et al. (44 patients from three institutions) evaluated a novel parameter, named total discordant volume (TDV), obtained by summing the volumes of mis-matched lesions among 44 individuals diagnosed with a GEP-NET. OS was longer in the low-TDV cohort than in the high-TDV cohort (median volume, 43.7 cm^3^; survival time, 23.8 months vs. 9.4 months; hazard ratio, 0.466 [95% CI, 0.229–0.948]; *P* = 0.0221) [74].

A proper standardization of the use of different tracers, and of the interpretation of their combined uptake profiles, will more accurately define tumor biology, allowing a tailored subsequent treatment choice.

### Enhancing PRRT: Combination Therapies

Since advanced-stage NEN frequently display inter-/intra-tumor heterogeneity, various combination therapies have been considered in addition to PRRT, to improve efficacy and target all components of the whole tumor burden.

As for all radiation treatments, the issues of radiosensitivity and radioresistance are worth of consideration. It is known that chemotherapy can work both with a direct cytotoxic effect and as a radiosensitizer, contrasting DNA repair and cell proliferation; this rationale led researchers to analyze the combination of various cytotoxic treatments with PRRT, obtaining promising results on the outcome and safety profile [75–77].

Nicolini et al. selected 37 patients with G1–G3 GEP-NET, with high uptake on both SST and FDG PET/CT, to combine PRRT with oral capecitabine, administered in between PRRT cycles. They found a median PFS of 31.4 months, with median OS not reached during the follow-up period of 38 months. Grade 3 or 4 hematological toxicity affected 16.2% of patients, diarrhea occurred in 5.4%, and asthenia in 5.4% of them, showing an acceptable safety profile [78]. This study, as well as those previously conducted by Kong et al. [79] and by Kashyap et al. [80] (both on the combination of PRRT with 5-FU), highlights the potential benefit of combined chemo-PRRT in patients with advanced GEP NET presenting with SST and FDG-positive PET/CT. In all of the cited works, chemotherapy was administered as low dose, solely for a radiosensitizer scope, as first defined by van Essen et al. in their pilot study [81].

Conversely, Parghane et al. employed a full dose of capecitabine-temozolamide (CAPTEM) in a “sandwich” scheme, administering chemotherapy between two [177Lu]Lu-DOTATATE cycles in 38 patients with SST and FDG-avid metastatic NET, obtaining favorable response rates in terms of estimated PFS rate ( 72.5%) and OS rate (80.4%) at 36 months (median PFS and OS not reached at a median follow-up of 36 months) [82].

Two parallel phase II randomized trials are focusing on the CAPTEM-PRRT ([177Lu]Lu-DOTATATE) combination versus CAPTEM alone in the treatment of pNET and versus PRRT alone in midgut neuroendocrine tumors (NCT02358356).

An alternative radiosensitizer method consists in contrasting the DNA repair mechanisms acted by tumor cells in response to radiotherapy. With this aim, some researchers employed poli-ADP ribose polymerase (PARP) inhibitors in combination with PRRT. Available data come mostly from preclinical studies [83, 84], but ongoing clinical trials are currently evaluating the use of olaparib (NCT04086485, NCT05870423, NCT04375267) and talazoparib (NCT05053854) in combination with PRRT.

Another approach to maximize PRRT efficacy is to hinder the neovascularization that frequently characterizes advanced tumors (including NEN), which tends to obstacle drug delivery, due to the fragile and disorganized blood vessels produced under the abnormal stimulation of VEGF. Sunitinib is a multi tyrosin kinase inhibitor with an anti-proliferative and anti-angiogenic action, only approved for the treatment of advanced pancreatic NET on the basis of the SUNNET phase III trial [85]. Its use in combination to PRRT as a radiosensitizer has been successfully explored [86, 87] and a trial is in progress (NCT05687123). The Occlurandom trial is currently investigating safety and efficacy of [177Lu]Lu-DOTATATE vs Sunitinib in unresectable progressive well-differentiated neuroendocrine pancreatic tumor (NCT02230176).

The efficacy and safety of 177Lu-Edotreotide PRRT in comparison with everolimus have been investigated in the COMPETE trial, actually completed but with results no yet presented, with PFS as a primary endpoint (NCT03049189). The COMPOSE trial (NCT04919226) is comparing 177Lu-Edotreotide with Everolimus or chemotherapy (folfox or captem) and it is still recruiting.

The association between PRRT and immune-checkpoint inhibitor nivolumab is being assessed in a phase II single arm trial, evaluating preliminary safety and efficacy of the combination in G3 NET and NEC (still recruiting, NCT04525638). Another clinical trial aims to evaluate the combination of pembrolizumab plus liver-directed therapy or PRRT (NCT03457948).

Another strategy that is currently under investigation is the upregulation of SST, as the cytotoxicity of PRRT strictly depends on SST density on the surface of tumor cells. Chemotherapy [88] and everolimus [89] were reported to promote the expression of SST and cold SST analogs were hypothesized to act in a similar way [90]. In addition, novel epigenetic drugs demonstrated the ability to successfully upregulate SST expression in vitro [6] and in vivo [91], potentially enabling PRRT in cases of initial low SST expression. Modulating SST is desirable to strengthen the therapeutic effect of PRRT, but the optimal approach to increase SST expression is not defined yet.

Lastly, the benefit of dual-PRRT, i.e., the combination of different PRRT pharmaceuticals, has been outlined by different studies [92, 93], and it is worthy of consideration for future trials.

The therapeutic options available to date for advanced NEN can target different molecular pathways in the absence of clear prioritization or established sequencing protocols. The studies conducted so far suggest that a synergistic effect with PRRT may be expected but robust evidence is still needed.

### Emerging Indications

The superiority of PRRT over cold somatostatin analogs as a second-line treatment for advanced G1 and G2 GEP-NET is well-accepted. Bringing PRRT forward to first line has been recently considered for metastatic higher-grade NET. Netter 2 study (NCT03972488) is a phase III trial that aims to evaluate the efficacy of first-line [177Lu]Lu-DOTATATE to treat advanced high-grade G2 and G3 (ki67 > 10%; < 55%) SST-expressing GEP-NET patients, comparing it to long-acting release octreotide (4 cycles of 7.4 GBq [177]Lu-DOTATATE vs 60 mg octreotide every 4 weeks). Preliminary results have recently been presented and showed a median PFS (95% confidence interval) of 8.5 months in the control arm and 22.8 months in the PRRT arm, the latter with limited toxicity (≤ 3 cases of grade 3–4 leukopenia, anemia, and thrombocytopenia and one case of MDS) [94••]. ESMO 2020 guidelines report that PRRT may be considered in patients with NET G3 [9]; however, patients need to be carefully selected and prospective trials are warranted to further establish which patients with NEN G3 might benefit most from PRRT. The above discussed NETTER-2 trial has recently been initiated to address this issue (NCT03972488). Preliminary data on PRRT in NEN G3 (*n* = 280pts in four retrospective studies) [95–98] reported disease control rates between 30 and 80% (PFS 9–23 months and OS 19–53 months).

Induction PRRT has been explored for unresectable tumors prior to surgery (i.e., neoadjuvant treatment) [99, 100] or followed by further maintenance cycles [101].

Minczeles et al. administered [177Lu]Lu-DOTATATE with the scope of downstaging in 49 patients with pancreatic NET. Of these, 26 underwent surgery with curative intent, obtaining a median OS of 14.7 years (95% CI 5.9–23.6), compared to 5.5 years (95% CI 4.5–6.5) for the PRRT-only group (*P* = 0.003). Median PFS was 5.3 years (95% CI 2.4–8.1) for the PRRT + surgery group and 3.0 years (95% CI 1.6–4.4) for the PRRT-only group (*P* = 0.02) [99].

A prospective on-going phase II single-arm trial (Neo.Lu.Pa.NET) aims to investigate evaluated safety and efficacy of neoadjuvant PRRT with [177Lu]Lu-DOTATATE followed by surgery for resectable pancreatic neuroendocrine tumors (NCT04385992).

### New Tracers and Theranostic Pairs

Fluorine-labeled somatostatin (SST) agonists have been proposed as an alternative to [68 Ga]-labeled PET tracers due to the advantages of [18F] in terms of longer half-life (110 min vs. 68 min), easier distribution to other centers, more favorable positron range (resulting in better image quality), higher activity amounts available per center (in contrast with the limited [68 Ga] doses obtainable per single elution). In a recent multicenter study, [18F]F-Alf-NOTA-octreotide showed an equal or superior [68 Ga]Ga-DOTATATE/NOC detection rate (91.1% vs. 75.3%; *P* < 10–5) [102], and the first clinical studies on [18F]F-SiFAlinTATE described favorable tumor-to-liver and tumor-to-spleen ratios [103].

SST antagonists display pharmacokinetic characteristics (low internalization, reduced dissociation, higher receptor binding) and low backgrounds that translate into superior detection rates compared to SST-agonists. A recent bi-center prospective study compared [68 Ga]Ga-NODAGA-JR11 and [68 Ga]Ga-DOTATATE in a cohort of 100 patients with unresectable or metastatic NET. Superior detection ability and superimposable lesion uptake were found for [68 Ga]Ga-NODAGA-JR11 compared to [68 Ga]Ga-DOTATATE, with the latter being superior only in detecting bone lesions (but without statistical significance and justified by the lower JR11 bone marrow uptake). Sensitivity was higher for [68 Ga]Ga-NODAGA-JR11 (91.7%, range, 87.6–95.7%) compared to [68 Ga]Ga-DOTATATE (77.2%, range, 71.0–83.4%), using conventional imaging as a reference [104]. [177Lu]Lu-DOTA-JR11, the corresponding therapeutic radionuclide, has been shown to cause more hematologic adverse events than [177Lu]Lu-DOTATATE. Reidy-Lagunes et al. have hypothesized that 177Lu-DOTA-JR11 could target SST expressed by stem cells or progenitor cells [105].

[68 Ga]Ga-NODAGA-LM3 and [68 Ga]Ga-DOTA-LM3 are novel antagonists with similar high tumor accumulation [106]. In a newly published paper, Liu et al. described the use of four types of antagonists ([68 Ga]Ga-NODAGA-LM3, [68 Ga]Ga-DOTA-LM3, [68 Ga]Ga-NODAGA-JR11, and [68 Ga]Ga-DOTA-JR11) in 549 patients, with 181 of them having a [68 Ga]Ga-DOTATATE PET/CT scan as comparison. The authors found that only [68 Ga]Ga-NODAGA-LM3 produced a significantly higher uptake than [68 Ga]Ga-DOTATATE in the hottest lesions (SUVmax = 57.4 ± 38.5 vs. 40.0 ± 22.8, *P* < 0.001), while the other antagonists showed comparable or lower SUVmax then [68 Ga]Ga-DOTATATE. All the antagonists displayed a higher TLR compared to [68 Ga]Ga-DOTATATE [107].

SST-targeting tracers labeled with alpha emitters [212Pb], [213Bi] [108], and [225Ac] [109] have been developed for their promising characteristics, i.e., higher linear energy transfer and lower tissue penetration (40–100 µm) compared to beta-emitters, allowing to deliver higher cytotoxic radiation to the tumor while sparing normal tissues.

[212Pb]Pb-DOTAM-TATE was well tolerated in phase I studies [110] and it is currently under evaluation for treatment-naïve NET patients (NCT03466216).

An interesting and emerging setting is the possbility to use alpha emitters for PRRT to treat relapsing patients after 177Lu-PRRT failure. The use of [225Ac]Ac-DOTATATE has been proposed for 177Lu-PRRT refractory patients or patients with PD after the first 177Lu-PRRT course: Ballal et al. administered [225Ac]Ac-DOTATATE in 91 patients with metastatic GEP-NET, 57 of them with prior [177Lu]Lu-DOTATATE treatment. Median OS and PFS were not reached during the 24-month follow-up. Two of 79 patients (2.5%) had CR, 38 (48%) had a PR, and 23 (29%) had SD. The treatment was safe, without higher grade toxicities [111]. The on-going trial ACTION-1 is also investigating the feasibility of [225Ac]Ac-DOTATATE following PRRT with [177Lu]Lu-labeled-PRRT failure (NCT05477576).

### Other Issues

Neuroendocrine liver metastases can be targeted with transcatheter intra-arterial liver-directed therapies, if surgery is not feasible.

Loco-regional treatments include embolization, chemoembolization, and radioembolization with [90Y]-glass/resin microspheres (preceded by [99mTc]-macroaggregates of albumin SPECT/CT for pre-treatment evaluation) or radioembolization with [166Ho]-microspheres (made of poly-l-lactic acid) [112]. Radioembolization with [166Ho]-microspheres presents the theranostic advantage of using a scout dose of [166Ho]-microspheres to safely evaluate their intra-arterial distribution before [166Ho]-microsphere treatment.

Intra-arterial (IA) PRRT has been proposed for NET patients with predominant liver involvement, hypothesizing a more favorable tumor uptake compared to the intra-venous (IV) administration of [177Lu]Lu-DOTATATE, thanks to the first-pass effect. Promising results were obtained through the comparison of IA and IV PRRT in different patient populations, with the limitations of the heterogeneous primary sites, SST expression, ki67, and previous therapies [113]. The LUTIA randomized controlled trial, conversely, was designed using an intra-patient control, injecting [177Lu]Lu-DOTATATE (4 cycles of 7.4 Gbq) in only one hepatic lobe, in 27 patients with bilobar NET liver metastases (G1 and G2 GEP-NET). The authors did not find significant differences between the tumor-to-non-tumor (T/N) uptake ratio of the two lobes on the 24 h post-treatment [177Lu]Lu-SPECT/CT after the first cycle, which was the primary endpoint (T/N IA = 17.4 vs. T/N control = 16.2; *P* = 0.299) [114]. Future trials are needed to evaluate this treatment strategy.

With respect to scheme personalization, Garske-Román et al. investigated a dosimetry-guided approach to allow the prolonging of PRRT cycles in patients that do not reach the limiting absorbed doses of 23 Gy to the kidneys or 2 Gy to the bone marrow within the standard 4 cycles of 7.4 GBq of [177Lu]Lu-DOTATATE each. They continued administering further cycles besides the first four in 200 patients, until achieving therapy-limiting doses. Results displayed that the individualized approach is feasible, with the limiting absorbed dose to the kidneys reached in 3 to 9 cycles in 123 patients and none experimenting the limiting dose to the bone marrow. Hematological toxicity regarded three patients (1.5%) with post-PRRT acute leukemia, and 30 (15%) grade 3 or 4 bone marrow toxicity. Eight patients (4%) had grade 2 kidney toxicity and one patient (0.5%) grade 4 kidney toxicity [115].

The value of personalized versus fixed schemes is under investigation (NCT03454763, NCT04917484).

## Conclusion

Theranostics is one of the most promising applications of precision nuclear medicine. In the setting of NET, the cellular target is represented by the SST overexpressed on tumor cells. Approved [68 Ga]-SST radiopharmaceuticals are routinely used for diagnostic PET/CT imaging to assess SST-expressing tumor burden and to assess eligibility for targeted therapy with the approved SST-targeted [177Lu]Lu-DOTATATE. PRRT is an effective treatment for well differentiated NET, already included in EANM/SNMMI, ESMO, and ENETS guidelines. Along with a favorable toxicity profile, it is associated with a strong impact on quality of life (longer time interval to quality of life deterioration and reductions of symptoms). To optimize the therapeutic index, many studies are on-going investigating delivery of PRRT at earlier time-points in the natural history of the disease, comparison with other approved drugs, combination with chemo/target therapy, PRRT re-treatment, different administering PRRT protocols (cycles number and dose/cycle), use and combination of different radionuclide therapies (beta-minus and alpha emitters), and development of new theranostic pairs.

## Data Availability

No datasets were generated or analyzed during the current study.
